# Using metabarcoding to compare the suitability of two blood‐feeding leech species for sampling mammalian diversity in North Borneo

**DOI:** 10.1111/1755-0998.12943

**Published:** 2018-10-16

**Authors:** Rosie Drinkwater, Ida Bærholm Schnell, Kristine Bohmann, Henry Bernard, Géraldine Veron, Elizabeth Clare, M. Thomas P. Gilbert, Stephen J. Rossiter

**Affiliations:** ^1^ School of Biological and Chemical Sciences Queen Mary University of London London UK; ^2^ Natural History Museum of Denmark University of Copenhagen Copenhagen Denmark; ^3^ School of Biological Sciences University of East Anglia, Norwich Research Park Norwich, Norfolk UK; ^4^ Institute for Tropical Biology and Conservation Universiti Malaysia Sabah, Jalan UMS Kota Kinabalu Sabah Malaysia; ^5^ Institut Systématique Evolution Biodiversité (ISYEB) Muséum National d'Histoire Naturelle, CNRS, Sorbonne Université, EPHE Paris Cedex France; ^6^ NTNU University Museum, Norwegian University of Science and Technology Trondheim Norway

**Keywords:** biodiversity, Borneo, Haemadipsidae, invertebrate‐derived DNA, mammals, metabarcoding

## Abstract

The application of high‐throughput sequencing (HTS) for metabarcoding of mixed samples offers new opportunities in conservation biology. Recently, the successful detection of prey DNA from the guts of leeches has raised the possibility that these, and other blood‐feeding invertebrates, might serve as useful samplers of mammals. Yet little is known about whether sympatric leech species differ in their feeding preferences, and whether this has a bearing on their relative suitability for monitoring local mammalian diversity. To address these questions, we collected spatially matched samples of two congeneric leech species *Haemadipsa picta* and *Haemadipsa sumatrana* from lowland rainforest in Borneo. For each species, we pooled ~500 leeches into batches of 10 individuals, performed PCR to target a section of the mammalian 16S rRNA locus and undertook sequencing of amplicon libraries using an Illumina MiSeq. In total, we identified sequences from 14 mammalian genera, spanning nine families and five orders. We found greater numbers of detections, and higher diversity of OTUs, in *H. picta* compared with *H. sumatrana*, with rodents only present in the former leech species. However, comparison of samples from across the landscape revealed no significant difference in mammal community composition between the leech species. We therefore suggest that *H. picta* is the more suitable iDNA sampler in this degraded Bornean forest. We conclude that the choice of invertebrate sampler can influence the detectability of different mammal groups and that this should be accounted for when designing iDNA studies.

## 1 INTRODUCTION

The rapid assessment of biodiversity through metabarcoding offers new opportunities in ecology. In particular, the ability to amplify and deep sequence the DNA from mixed sources contained within environmental samples has led to renewed interest in applying noninvasive molecular techniques to address questions in conservation. DNA metabarcoding is now a common technique to catalogue diversity (Deiner, Fronhofer, Mächler, Walser, & Altermatt, 2016) and infer species interactions, including trophic connections (Salinas‐Ramos, Herrera Montalvo, León‐Regagnon, Arrizabalaga‐Escudero, & Clare, 2015).

One application of DNA metabarcoding that has shown particular promise for biodiversity monitoring is the screening of invertebrate‐derived DNA (iDNA). Early research using iDNA techniques often had an epidemiological focus, for example, screening insect vector bloodmeals to identify hosts (review by Kent, 2009). More recently, these molecular techniques have been applied to biodiversity monitoring, including species of conservation concern (Schnell et al., 2012). A small number of studies have identified vertebrates from DNA contained within the blood meals of haematophagous (blood‐feeding) leeches (Schnell et al., 2012; Weiskopf et al., 2017), while others have targeted blood or wound feeding arthropods including blowflies (Calvignac‐Spencer, Merkel, et al., 2013; Lee, Sing, & Wilson, 2015), mosquitoes and sand‐flies (Kocher, Thoisy, Catzeflis, Huguin, et al., 2017). Sources of iDNA are not only restricted to blood; indeed, host DNA can be also recovered from invertebrate taxa that feed on faeces (Gómez & Kolokotronis, 2016), and potentially from other excreta (see review by Calvignac‐Spencer, Leendertz, Gilbert, & Schubert, 2013).

Mounting interest in the potential use of invertebrates as samplers stems in part from falling sequencing costs in addition to a number of perceived advantages over more traditional methods (also see Weiskopf et al., 2017). For example, field sampling of invertebrates is often logistically easier and cheaper and tends to result in a greater number of individuals than direct or indirect sampling of vertebrates, including live trapping (Wells, Pfeiffer, Lakim, & Linsenmair, 2004) or camera trapping (Wearn, Rowcliffe, Carbone, Bernard, & Ewers, 2013). Sampling of vertebrates is also more tightly regulated than that of invertebrates, with stricter laws governing ethical handling and the transport of material across international borders (Sikes & Gannon, 2011). Furthermore, using iDNA removes the need for field‐based taxonomic expertise as species identification can be achieved after sequencing with bioinformatics (Wheeler, Raven, & Wilson, 2004).

Despite the interest among ecologists in using iDNA for sampling, this approach still requires development and many aspects regarding its utility have not been fully addressed. For iDNA monitoring programmes to be successful, we need a deeper understanding of how the choice of an invertebrate sampler might influence biodiversity estimates for a given ecosystem at a local scale. Multiple aspects of the invertebrate's biology will likely affect vertebrate detection probabilities (Calvignac‐Spencer, Leendertz, et al., 2013). For example, variation in dispersal behaviour, habitat‐use, feeding ecology and rate of digestion should all ideally be taken into account when choosing a sampler species (Schnell, Sollmann, et al., 2015). Other biases are known to arise from the laboratory protocols, although these have been examined previously, and are better understood than those biases introduced by the invertebrate sampler (Alberdi, Aizpurua, Gilbert, & Bohmann, 2017; Elbrecht, Peinert, & Leese, 2017).

Terrestrial leeches (family Haemadipsidae, phylum Annelida) are free‐living blood‐feeders, with ~50 species, distributed across the palaeo‐tropics (Sket & Trontelj, 2008). Apart from being highly abundant and easy to collect, haemadipsid leeches may be particularly useful as iDNA sources because of their large body size and gut capacity compared with most blood‐feeding arthropods (Schnell, Sollmann, et al., 2015). In addition, the use of leech iDNA has been shown to be a complementary method to camera trapping (Weiskopf et al., 2017). Most common haemadipsid leeches have been suggested to be generalist feeders, opportunistically attaching to passing mammalian hosts (Govedich, Moser, & Davies, 2004), and this has received recent support from wide‐scale sampling. Schnell et al. (2018) compared haemadipsid leeches across five geographical regions and found evidence that across the family, species feed on a broad range of mammalian diversity, while Tessler et al. (2018) reported similar trends from three additional regions. However, finer‐scale comparisons of iDNA samplers from the same site (Kocher, Thoisy, Catzeflis, Valiere, et al., 2017) have rarely been undertaken.

Here, we perform a quantitative comparison of diet of two co‐occurring terrestrial leeches *Haemadipsa sumatrana *(commonly called the brown leech) and *Haemadipsa picta *(the tiger leech) to test their relative usefulness as samplers of a diverse mammal fauna in lowland tropical forest in Borneo, South‐East Asia. Although these two species occupy the same forests, they appear to have different fine‐scale habitat associations. *H. sumatrana is *found almost exclusively in the leaf litter, while *H. picta *is found from the ground to around two metres in the understorey (Lai, Nakano, & Chen, 2011). *H. picta* also appears to be robust to microclimatic changes; they are more common than *H. sumatrana* in logged forest with open canopy (Kendall, 2012) and are also more likely to occur on or near trails in the forest (Gąsiorek & Różycka, 2017). Other unknown species‐specific traits may also contribute to differences in their feeding ecology.

Our specific aims were to (a) use metabarcoding techniques to ascertain the feeding ecology of both leech species, (b) compare the diets of spatially matched leeches across a range of forest types and (c) evaluate the suitability of using each of the leech species as an iDNA sampler for biodiversity monitoring. Being able to rapidly identify and understand differences in mammalian diversity in Bornean forests is especially pertinent in the light of ongoing land‐use change. Specifically, forest outside of protected areas is often highly degraded due to timber extraction and conversion for agriculture (Gaveau et al., 2014). Furthermore, large vertebrates, such as charismatic and rare large mammals, are key considerations in the formulation of policy or conservation actions and decision‐making will rest on the assumptions of data reliability.

## 2 MATERIALS AND METHODS

### 2.1 Study site and sample collection

**Figure 1:**
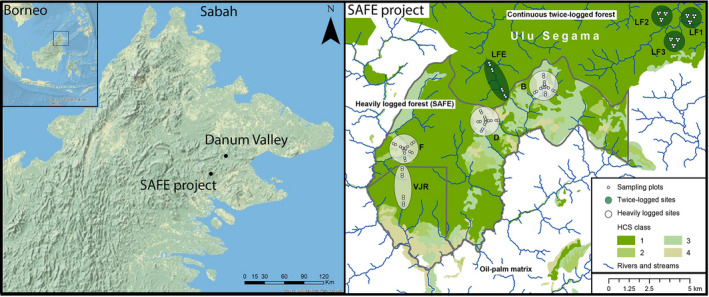
Maps showing the location of the Malaysian state of Sabah in North Borneo, with the locations of the SAFE project (Ewers et al., 2011) and Danum Valley (protected area ‐ primary rainforest). The right panel is a detailed map of the sampling at the SAFE project. Green circles are the continuous logged forest blocks and have been twice‐logged, whereas the white circles are the degraded forest blocks and have been heavily logged within the SAFE project experimental area. Small circles show the location of the vegetation plots where the sampling took place. Blue lines represent the rivers and streams, and forest is coloured according to the classification of high carbon stock forest (HCS)

We collected all samples at the Stability of Altered Forest Ecosystems (SAFE) site in the Kalabakan Forest Reserve, Sabah (4° 33′ N, 117° 16′ E) in Malaysian Borneo, a large‐scale forest fragmentation experiment covering logged secondary forest (Ewers et al., 2011). There are eight forest blocks (3 km^2 ^radius) that we broadly classified as either degraded (*n* = 4) or continuous logged forest (*n* = 4) (Figure 1). Degraded forest blocks consisted of heavily logged and fragmented forest patches (including the large VJR fragment) found within an oil‐palm matrix, whereas the continuous logged forest blocks consisted of twice‐logged forest located within a large contiguous tract of managed forest (Ewers et al., 2011). Each of these blocks contains between 8 and 16 permanent forest plots (25 m^2^) (for details, see Ewers et al., 2011). We collected samples of leeches from 59 forest plots in degraded forest (Figure 1; blocks B, D, F & VJR) and 29 plots in continuous logged forest (Figure 1; blocks LF1–3 & LFE). Forest plots were selected based on accessibility and microclimatic conditions that support leech populations.

Within each plot, leeches were sampled by searching the forest floor and understorey for 20 min, and each plot was resampled four times between February and June 2015. All leeches encountered of both species, *H. picta* and *H. sumatrana*, were collected and placed into individual tubes containing RNA later. The samples were stored on ice packs in cool boxes until returning to the main camp, normally within 12 hr but for some remote sites the delay was 3–4 days. *H. picta* and *H. sumatrana *were the only leech species encountered in the field and were easily identifiable based on their markings.

### 2.2 DNA extraction and PCR amplification

**Figure 2:**
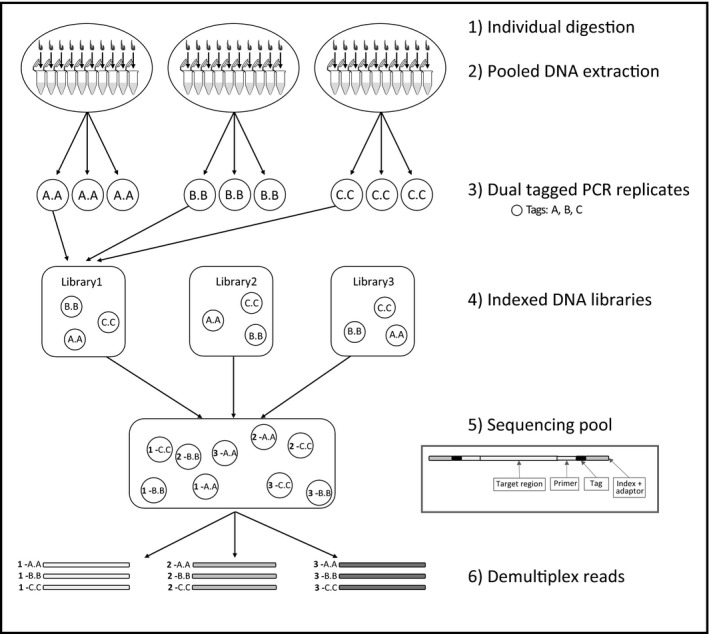
Workflow diagram for pooled terrestrial leech iDNA sequencing; (1) individual leeches are digested, (2) DNA is extracted from batches of 10 individual leech digests, (3) PCR replicates are uniquely dual‐tagged (e.g., A.A), (4) PCR replicates are pooled for DNA library build with unique tag/index combinations, (5) libraries are mixed in equimolar concentration and sequenced, (6) sequences are demultiplexed by index and sorted by PCR replicate. Insert shows magnification of an amplicon with the tag and index order

We performed sequencing of pooled leeches following the protocol set out by Schnell et al. (2018) (Figure 2). Briefly, we performed tissue digestions on individual leeches using the tissue digestion buffer with enough buffer per leech to equal a volume approximately five times the leech body and then incubated the samples overnight at 50°C while gently shaking. Following this incubation, we pooled 100 µl of 10 individual digests, ensuring that each 1,000 µl pool contained 10 leeches collected from the same block (Figure 1). In total, 490 *H. sumatrana* were pooled into 49 pools and 520 *H. picta* were pooled into 52 pools (Table 1). For each leech digest pool, we purified the DNA using the QiaQuick DNA kit (Qiagen, UK) following the manufacturer's protocols but with a modified centrifugation procedure (1 min at 6,000 *g*, 1 min at 10,000 *g* followed by an additional 3 min at full speed and 1 min at 12,000 *g*) and eluted in 50 µl EB buffer. To ensure consistency, for each batch of extractions, we quantified the DNA from a subsample of pools using the Qubit dsDNA HS Assay Kit (Invitrogen).

**Table 1:** Summary of leech samples pooled, extracted and sequenced for both species (*Haemadipsa sumatrana *and *Haemadipsa picta*) and where they were collected from

Block	Habitat type	Samples extracted (pools)		Samples sequences (pools, individuals)	
		*H. sumatrana*	*H. picta*	*H. sumatrana*	*H. picta*
B	Degraded	8	11	6, 60	10, 100
D	Degraded	0	4	0, 0	3, 30
F	Degraded	18	4	13, 130	4, 40^a^
VJR	Degraded	2	1	2, 20	1, 10
LFE	Continuous logged	13	19	12, 120	18, 180
LF1	Continuous logged	1	0	1, 10^a^	0, 0
LF2	Continuous logged	0	3	0, 0	3, 30^a^
LF3	Continuous logged	7	10	7, 70^a^	10, 100^a^

a Samples which only had duplicate PCR replicates

We amplified a 95 bp fragment of the mammalian 16 s mitochondrial gene using the primers “16Smam1” forward 5′‐CGG TTG GGG TGA CCT CGGA‐3′ and “16Smam2” reverse 5′‐GCT GTT ATC CCT AGG GTA ACT‐3′ primers (Taylor, 1996). We conducted PCRs in triplicate, with the exception of 26 pools (from LF1–3 & F) which were conducted in duplicate during a preliminary experiment (Table 1). DNA was amplified using 5′ nucleotide tagged primers (6–8 bp) (Binladen et al., 2007), with identical tags on both forward and reverse primers to be able to identify possible errors due to “tag jumping” (Schnell, Bohmann, & Gilbert, 2015). All tags were designed to have two mismatches between each pair, to allow for identification in the case of sequencing error (Binladen et al., 2007). The 25 µl PCR products consisted of 0.2 mM of 10× buffer, 2.5 mM MgCl_2_, 1 unit DNA polymerase (AmpliTaq Gold, Applied Biosystems), 0.2 mM dNTP mix (Invitrogen), 0.5 mg/ml BSA, 0.6 µM of each primer and 1 µl of DNA template and with a thermal cycling profile of 95°C for 5 min, then 40 cycles of 95°C for 12 s, 59°C for 30 s and 70°C for 20 s with a final extension time of 7 min at 70°C. Negative extraction, PCR and positive controls (giraffe DNA) were included in every run. All PCR products (including controls) were visualized on 2% agarose gels, and those reactions which contained DNA were pooled into libraries. PCR success rate was high, with 94% of *H. picta* pools and 83% of *H. sumatrana* pools seen to contain vertebrate DNA. Using these successful PCR replicates (including controls), we prepared indexed amplicon libraries for sequencing using the best v2.0 library build protocol (Carøe et al., 2017). All amplicon libraries were checked pre‐ and postindexing using the 2100 Bioanalyzer, DNA high sensitivity kit (Agilent, Denmark). We pooled all indexed amplicon libraries at equimolar concentrations for sequencing on an Illumina MiSeq. Most libraries were sequenced (250 bp paired‐end) at the National High‐throughput DNA Sequencing Centre (University of Copenhagen) with a smaller number sequenced (150 bp paired‐end) at the Bart's and the London Genome Centre (Queen Mary University London).

### 2.3 Bioinformatics and statistical analyses

We merged demultiplexed forward and reverse reads using adapterremoval v2 (Schubert, Lindgreen, & Orlando, 2016) with default parameters except for minalignment 100, minlength 50 and shift 5. On the merged reads, we used a modified version of dame (https://github.com/shyamsg/DAMe, Zepeda‐Mendoza, Bohmann, Carmona Baez, & Gilbert, 2016) to assign each sequence to the original sample based on the correct primer and nucleotide tag combination. With dame, we retained only those sequences that were detected in a minimum of two replicates, clustered the filtered reads at 97% similarity using sumaclust v1.3 (Mercier, Boyer, Bonin, & Coissac, 2013) and normalized the reads per sample to 50,000 with dame to allow cross‐sample comparisons. To identify potential sequencing errors, a postclustering filtering procedure was then applied to the original OTU table using LULU, which removes erroneous rare OTUs based on both sequence similarity thresholds and within‐sample patterns of co‐occurrence (Frøslev et al., 2017). We detected some evidence of contamination in the negative controls with sequences matching two OTUs, corresponding to *Rusa unicolor* and *Sus barbatus,* respectively. For the remaining samples, we therefore only considered these species to be present where numbers or reads exceeded those found in the controls.

### 2.4 Compiling the reference database

Using local knowledge of the field site, field guides (Payne, Francis, & Phillipps, 1985; Phillipps & Phillipps, 2016) and the IUCN red list distribution maps (www.iucnredlist.org), we complied a reference database of all mammals likely to occur at the study site. We retrieved all published 16S from NCBI GenBank nucleotide database, aiming for five records per species (Supporting Information Figure S1). We trimmed and aligned the selected sequences to our target region using aliview (Larsson, 2014). To augment our database, we generated new 16S sequences for the following species: Common treeshrew (*Tupaia glis*)*, *Small‐toothed palm civet (*Arctogalidia trivirgata*), Banded civet (*Hemigalus derbyanus*), Common palm civet (*Paradoxurus hermaphroditus*)*, *Hose's civet (*Diplogale hosei*)*, *Malay civet (*Viverra tangalunga)*, Banded linsang (*Prionodon linsang*)*,* Short‐tailed mongoose (*Urva brachyura*), Collared mongoose (*Urva semitorquata*)*,* Chinese ferret‐badger (*Melogale moschata*), Malay weasel (*Mustela nudipes*) and Clouded leopard (*Neofelis nebulosa*) (Supporting Information Table S1). To be able to identify contamination, 16S sequence records from NCBI GenBank database were also included for human, giraffe (positive control) and domestic/human‐associated species (Supporting Information Table S2). Representative 16S sequences for reptiles, amphibians and birds were also included in the reference database (Supporting Information Table S2), sourced from NCBI GenBank, as detection of these taxa with iDNA by haemadipsids is known (Schnell et al., 2018; Tessler et al., 2018).

### 2.5 Taxonomic assignment

To assign each OTU to a mammalian taxon, we performed a blast search against a custom sequence reference database for Bornean taxa. We used the megan lowest common ancestor (LCA) algorithm to assign mammalian taxon to the OTUs from the top blast results with >90% similarity. We used the megan parameters of minimum bit score = 150, top per cent = 2, min support = 1 and weighted LCA with 90% coverage (Huson, Auch, Qi, & Schuster, 2007). We only considered assignments at the genus‐level, as this has been shown to increase reliability of identifications in other ribosomal markers when reference databases are incomplete (Kocher, Thoisy, Catzeflis, Huguin, et al., 2017). In a small number of cases where the best matching species (>90% similarity) has no known congeners in Borneo, we were able to assign to the species level (e.g., *Echinosorex gymnura*). Where there was no match to a reference sequence, the OTU remained unassigned and was removed from the analysis. We then filtered our results by removing any OTUs with a match to human or our positive control. Final taxonomic assignments are presented in Table 2.

**Table 2:** Taxonomic identity of mammal OTUs. The level of confidence in each assignment is shown by the bit score (megan) and the % identity match (blast). If two OTUs shared the same taxonomic identity values for both are given

Common name	Order	Family (subfamily)	Taxa assigned	OTU	% Identity	Bit score
Unknown deer	Artiodactyla	Cervidae (Cervinae)	Cervinae	OTU18	97	143
Sambar deer	Artiodactyla	Cervidae	*Rusa unicolor*	OTU4	100	171
Muntjac	Artiodactyla	Cervidae	*Muntiacus* sp.	OTU5/OTU7	91/90	159/154
Bearded pig	Artiodactyla	Suidae	*Sus barbatus*	OTU2	100	174
Mousedeer	Artiodactyla	Tragulidae	*Tragulus* sp.	OTU6	99	171
Banded civet	Carnivora	Viverridae (Hemigalinae)	*Hemigalus derbyanus*	OTU8	100	178
Malay civet	Carnivora	Viverridae (Viverrinae)	*Viverra tangalunga*	OTU12	100	178
Moonrat	Eulipotyphla	Erinaceidae	*Echinosorex gymnura*	OTU10	100	171
Macaque	Primate	Cercopithecidae (Cercopithecinae)	*Macaca* sp.	OTU9/OTU29	95/97	141/154
Leaf monkey	Primate	Cercopithecidae (Colobinae)	*Trachypithecus* sp.	OTU49	79	60
Gibbon	Primate	Hylobatidae	*Hylobates* sp.	OTU21	79	60
Hystrix porcupine	Rodentia	Hystricidae	*Hystrix* sp.	OTU13/OTU71	91/97	167/148
Long‐tailed porcupine	Rodentia	Hystricidae	*Trichys fasciculata*	OTU15	90	161
Rat	Rodentia	Muridae	*Rattus* sp.	OTU86	99	163

### 2.6 Estimation of biodiversity determined by leech samplers

To determine the relative utility of using the two focal leech species for iDNA sampling, we produced sample size‐based diversity accumulation curves using all samples together and compared these to leech species‐specific curves. To give a deeper understanding of the effects of rare and abundant taxa, we produced these curves by estimating three orders of Hill numbers of diversity (Chao et al., 2014). These are the most commonly used Hill numbers and are equivalent to species richness (*q* = 0), the exponential of Shannon–Weiner index (*q* = 1) and the Simpson diversity (*q* = 2). One of the benefits of using Hill numbers compared to other diversity indices is that they can be expressed in the terms of effective number of species, thus allowing different communities to be directly compared (Chao et al., 2014). In practice, this means a mammal community sampled by *H. picta* is comparable to a community sampled by *H. sumatrana*. For our accumulation curves, we used rarefaction to construct 84% confidence intervals (CIs) that equate to an *α*‐level of 0.05 for overlapping distributions, rather than 95% CIs that equate to an *α*‐level of 0.01 and are thus considered overly conservative in such comparisons (MacGregor‐Fors & Payton, 2013). Diversity accumulation curves with the standard 95% CI are shown in Supporting Information Figure S2.

To test whether leech species differ in their utility as iDNA samplers, we used two approaches. First, we fitted a GLM in which we modelled the number of detections per leech pool as the response variable with Poisson error, and fitted leech species (*H. picta* and *H. sumatrana*), forest type (degraded and continuous logged) and block identity (B, D, F, VJR, LF1, LF2, LF3, LFE) as explanatory variables. We started with a full model containing all variables and compared its fit based on AIC to seven reduced models (Supporting Information Table S3). Models showed no overdispersion (*θ* < 2). Second, to test whether the diets of two leech species differ with respect to composition of mammals, we examined patterns of beta‐diversity among pools. We calculated pairwise Bray–Curtis dissimilarity indices and visualized community composition using nonmetric multidimensional scaling (NMDS). To test for greater dissimilarity between leech species and different habitats, we applied a PERMANOVA analysis as a robust test of ecological community structure (Anderson & Walsh, 2013). We ran all analyses in r (R Core Team, 2018) using vegan (Oksanen et al., 2017) and inext packages (Hsieh, Ma, & Chao, 2016).

## 3 RESULTS

### 3.1 Generation of reference database

Our final reference database of sequences compiled for the field site contained 256 records of the 16S target sequence, from 28 mammalian families across ten orders. For 40 mammal species for which 16S sequences were not available, we either obtained published sequences from a related member of the same taxonomic family (30 cases), or we generated new sequences for Bornean native species (eight cases), or an Asian sister species (two cases: clouded leopard and Chinese ferret‐badger), via Sanger sequencing (Supporting Information Table S1). In this latter case, sequences ranged from 90 to 101 bp (new GenBank Accession nos MG996889–MG996900) (Supporting Information Figure S1).

### 3.2 Taxonomic assignment

By curating the total number of OTUs with the postclustering algorithm (LULU) and filtering out contaminants, we reduced the number of clustered OTUs from 65 to 17 (26% retained) but with no loss of taxonomic diversity. All OTUs matched to native Bornean mammalian taxa, and we found no unexpected taxa in our results. Of the 17 OTUs, 14 matched with high similarity to the reference sequences with >90% similarity (Table 2). Two of the remaining OTUs (OTU49 and OTU21) matched less well to a reference sequence (both at 79%) but were consistently assigned to langur (Colobinae) and gibbon (Hylobatidae), respectively. We also found one OTU with a match that could not be resolved beyond the subfamily Cervinae, matching equally to both the cervid genera that occur at the site (*Muntiacus* and *Rusa*).

**Figure 3:**
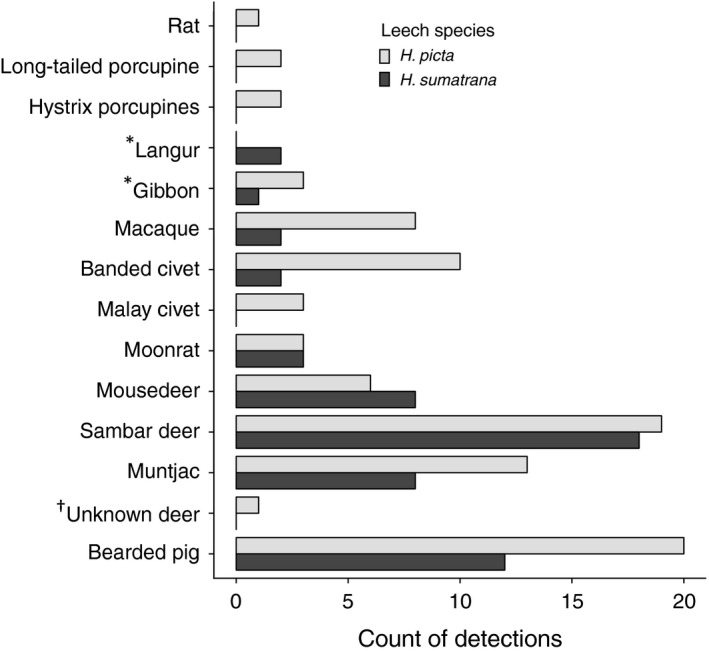
Comparison of the overall counts of different mammals identified using tiger leech samplers (*Haemadipsa picta*, light grey) compared with brown leech samplers (*Haemadipsa sumatrana*, dark grey). ^*^indicates the two cases where the sequence has a poor but consistent match to the reference database (<90% identity). ^†^indicates the sequence with the best match to both cervid genera (Rusa and Muntiacus)

Eight mammalian taxa were common to both leech species (Figure 3), of which the most prevalent was the Bornean sambar deer (*Rusa unicolor*) and bearded pig (*Sus barbatus*), followed by the muntjac *Muntiacus* spp. and the mousedeer *Tragulus* spp. (Figure 3). Other taxa were detected in both leech species but with considerably fewer detections in the brown leech (*H. sumatrana*) than in the tiger leech (*H. picta*): banded civet (*Hemigalus deryanus*), moonrat (*Echinosorex gymnura*), macaque (*Macaca* spp.) and gibbon (*Hylobates* sp.). Additionally, we found four taxa in the tiger leech that were not found in the brown leech: the Malay civet (*Viverra tangalunga*) and three rodents (two porcupine genera, *Hystrix* and *Trichys* as well as one *Rattus* sp). Finally, the langur (Colobinae) was only detected in the brown leech.

### 3.3 Mammal diversity in leech diet

**Figure 4:**
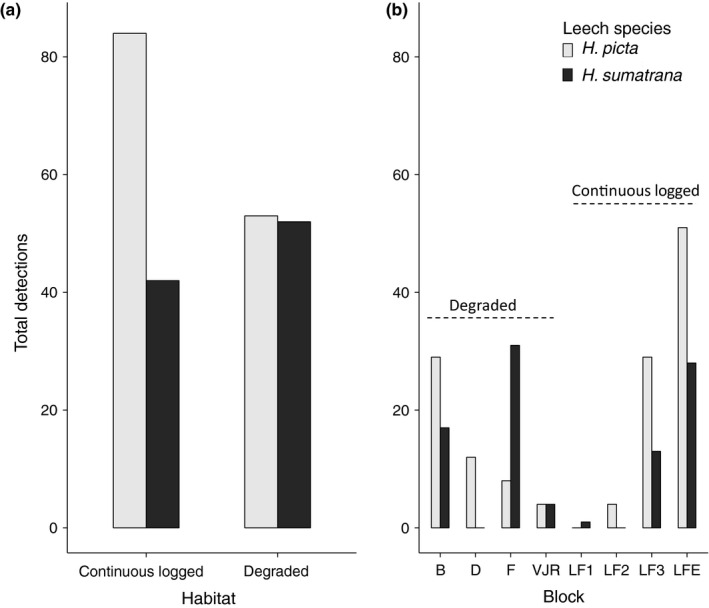
Total count of mammal detections found in (a) each habitat type and (b) at each block when sampling using each leech species (*Haemadipsa picta*, light grey and *Haemadipsa sumatrana*, dark grey)

We found a greater total number of detections in continuous logged forest in *H. picta* than *H. sumatrana* but very similar detection levels for both leech species in the degraded forests (Figure 4a). These trends were also reflected in most of the individual blocks sampled (Figure 4b). The results of the GLM indicated that number of mammal detections per pool was determined by leech species, with more detections in *H. picta*, but not by either habitat type or block (Table 3). Model comparisons suggested that the two best‐fitting models, each with similar AIC values, contained leech species alone (*F*_2,88_ = 20.86, *p* < 0.05) and leech plus habitat type (*F*_1,88_ = 30.28, *p* = 0.202). However, while the latter model was associated with the best fit (adj‐*R* ^2^ = 0.31), leech was the only significant single predictor (Table 3). Considering taxonomic representation, *H. picta *samples a greater proportion of orders (5/5), families (8/9) and genera (12/14) detected in this study compared with *H. sumatrana* (orders = 4/5, families = 7/9 and genera = 9/14). Of the species which could be identified, *H. picta *detects all six representatives, while *H. sumatrana* detects four.

**Table 3:** Summary output of the two final models (GLM with Poisson errors), to test the effects of the leech sampler species and habitat type on the number of detections

	Model 1	AIC = 295.65	Adj‐*R* ^2^ = 0.31	Model 2	AIC = 294.42	Adj‐*R* ^2^ = 0.25
	Parameter estimates (±*SE*)	*F*	*p*‐Value	Parameter estimates (±*SE*)	*F*	*p*‐Value
Intercept	0.77 (0.086)	79.62	>0.05*	0.83 (0.070)	138.58	>0.05*
Leech species	0.22 (0.092)	5.43	0.022*	0.20 (0.091)	4.69	0.032*
Habitat type	0.12 (0.092)	1.65	0.202			

*indicates significant test statistics at 0.05.

### 3.4 Accumulation of taxonomic richness

**Figure 5:**
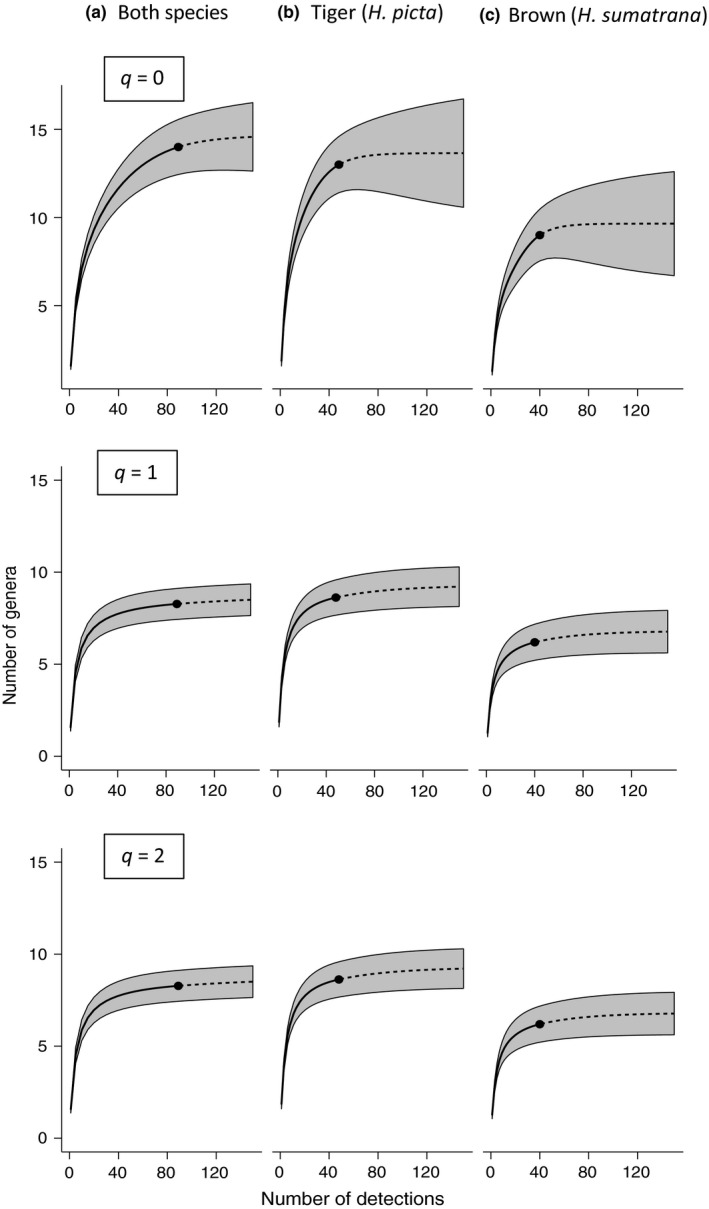
Diversity accumulation for: (a) both species together, (b) tiger leeches only (*Haemadipsa picta*) and (c) brown leeches only (*Haemadipsa sumatrana*). Each row shows the accumulation of mammal genera estimated for three hill numbers, *q* = 0, 1, 2. Curves are presented with 84% confidence intervals and extrapolated to 125 samples (dashed lines) following Chao et al. (2014)

Accumulation curves based on three metrics of mammalian diversity (equivalent to raw species richness, Shannon–Weiner index and Simpson diversity) showed consistent differences among the leech species. In each case, tiger leech consistently sampled around 40% more diversity than did the brown leech (Figure 5). Comparing these accumulation curves to corresponding curves based on pooled leeches suggested that *H. sumatrana* contained a subset of taxa of *H.* *picta*, with almost no additional diversity obtained by combining data from both leeches over that recorded for *H. picta* alone.

### 3.5 Estimates of local biodiversity between samplers

**Figure 6:**
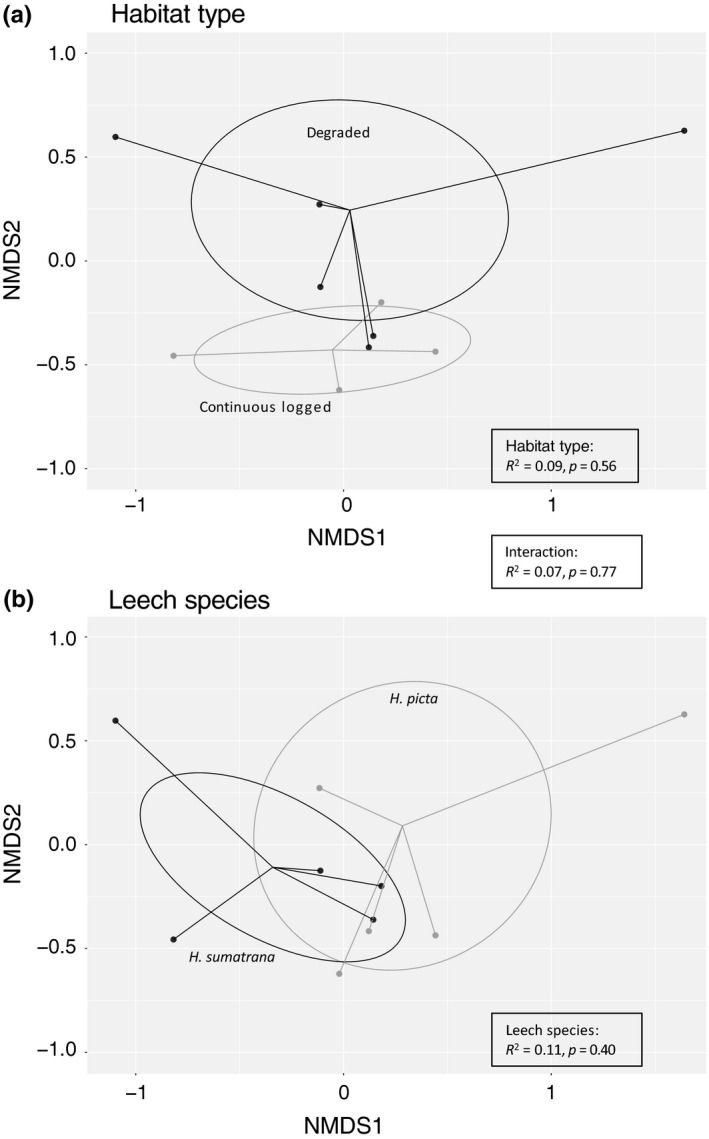
NMDS plots grouped by (a) habitat types and (b) leech species. Stress of the NMDS plots = 0.05, ellipses represent the standard error of the ordination. Values are the parameter estimates from the PERMANOVA test showing the nonsignificance between the groupings and the interaction

Visualizing the differences in community composition using NMDS showed see some separation between the community of mammals detected in the two habitat types, continuous logged and degraded forest (Figure 6a). When the data points were grouped by leech species, we found considerable overlap in the mammal communities sampled (Figure 6b). Despite some apparent separation with habitat, our PERMANOVA analysis found no significant difference between either of the factors or their interaction (Figure 6).

## 4 DISCUSSION

The use of invertebrates as iDNA samplers of vertebrates is gaining interest, and here, we have systematically assessed the relative utility of two congeneric haemadipsid species, tiger (*H. picta*) and brown (*H. sumatrana*) leech for detecting local mammal diversity. Using spatially matched samples for the detection of mammals in a degraded forest habitat in North Borneo, South‐East Asia, we analysed over 1,000 individual leeches from the two species, sampled at 88 sites across the landscape and we revealed the presence of terrestrial mammal species from nine families spanning five orders.

### 4.1 *Leech‐derived iDNA from H. picta *versus* H. sumatrana*

We found a high degree of overlap in the mammalian species richness detected in both *H. picta* and *H. sumatrana *diets. The most abundant detections correspond to the large common species found in the area such as sambar deer and bearded pig. However, of these leech species, *H. picta *has a significantly higher detection rate compared to *H. sumatrana*. There are nine overlapping mammal taxa in both species, but four taxa were only specific to *H. picta*, including all of the rodents detected. Sampling with *H. picta* results in a greater coverage of the total mammal community. By directly comparing the accumulation curves for a fixed sampling effort, that is, same number of equally sized leech pools, we would anticipate the detection of a greater diversity of effective numbers of species using *H. picta* compared to *H. sumatrana*. However, while we find greater abundance of detections, we did not detect a significant difference in the beta‐diversity of the mammal communities using either leech sampler.

Feeding strategies have been suggested to affect iDNA detection (Schnell, Sollmann, et al., 2015), and *H. picta* and *H. sumatrana *show clear differences in their searching and feeding behaviour; *H. sumatrana *is almost exclusively found at ground level and is camouflaged in the leaf litter, whereas *H. picta *tends to wait on leaves in the undergrowth and thus together with its more striking markings is easier to see and collect during sampling (Fogden & Proctor, 1985). Taking these points together, we suggest that of the two species examined, *H. picta* represents the more suitable iDNA sampler in our study area, due to the greater abundance of positive detections coupled with favourable behavioural traits for rapid sampling.

### 4.2 Detection of mammalian diversity

Although medium to large mammals, especially ungulates, were well represented in the sequence data, it was noticeable that very few small mammals were detected. In particular, nonvolant mammals from the three families, Tupaiidae (treeshrews), Sciuridae (squirrels) and Muridae (mice and rats) were not detected in any of the leech samples and yet are known to occur in the study area (Wearn et al., 2017). While our study is based on presence‐only data and, thus, nondetection cannot be used to infer absence from the habitat, it is noteworthy that a similar lack of Bornean small mammals was also recently reported by Schnell et al. (2018). These authors compared iDNA from leech bloodmeals sampled from a broad geographical scale and were able to detect treeshrews, squirrels and murid rodents in mainland South‐East Asia, Madagascar and Australia, but not in leech samples from Borneo. In addition, representatives of rodents and treeshrews were detected in a study of 200 leeches from Bangladesh based on Sanger sequencing of the 16S rRNA marker (Weiskopf et al., 2017). A recent study by Tessler et al. (2018) also confirmed detections of these small mammal groups in China and Bangladesh. The absence of these taxa from Bornean leeches might indicate that haemadipsid leeches in Borneo are behaving differently to their congeners in other parts of Asia.

As small mammals form a large part of the mammalian biomass in Borneo and a rich diversity of species are known to occupy the forest, the lack of detection specifically in Borneo is intriguing. Our reference database contained several representative sequences from all the small mammal families, so this is unlikely to be a consequence of missing reference data but in fact could reflect size‐related feeding preference shown by Bornean haemadipsids in particular. Whether or not leeches actively prey on large mammals or are more easily detected and ejected by small mammals is not known. Moreover, the underrepresentation of nocturnal mammals, such as murid rodents, cannot be explained by the timing of our surveys which were conducted during the day, since both of our focal leech species are active during both day and night. Regardless of the underlying causes of the observed patterns of detection, our data indicate that for the purposes of biodiversity surveys, Bornean leeches appear not to passively sample all nonvolant mammals in their environment, as has been previously suggested. Thus, we recommend that future iDNA studies should include assessments of how the ecology and behaviour of the chosen invertebrate sampler might influence any results.

### 4.3 Imperfect detections and temporal resolution

A major issue with the use of iDNA for biodiversity monitoring is imperfect species detection resulting from problems of false detections, both positive and negative. This is discussed in the wider literature concerning environmental DNA (eDNA) (Deiner et al., 2017; Roussel, Paillisson, Tréguier, & Petit, 2015) and also for iDNA studies (Schnell, Sollmann, et al., 2015). We have taken many steps to optimize the trade‐off between false positives and negatives, for example, using technical replicates to increase detection rates and reduce false negatives (Ficetola, Taberlet, & Coissac, 2016) while removing spurious tag combinations (Schnell, Bohmann, et al., 2015) and using conservative bioinformatic filtering to reduce false positives (Alberdi et al., 2017).

Imperfect detection of iDNA is also likely to be influenced by aspects of the biology of the sampler. Indeed, the rate of digestion of the blood meal and intervals between feeding events will affect the window of DNA detection (Schnell, Sollmann, et al., 2015). For the medicinal leech (*Hirudo medicinalis*), the detection window has been empirically tested and shown to be at least 120 days for mammal DNA (Schnell et al., 2012), and up to 50 days for mammalian viral DNA (Kampmann et al., 2017). However, *H. medicinalis* is a larger‐bodied taxon than *Haemadipsa *spp., with different ecology and behaviours, and the detection window remains unknown for our focal leech species. We do not know the temporal resolution of our mammal detections, and while this may be shorter than 120 days for *Haemadipsa* spp., we only retain the most abundant OTUs per sample (removal of singletons, etc), and this is likely going to limit vertebrate detection to the most recent blood meal only and, potentially, standardize the time frame of our detections between leech pools.

Previous studies have used several different molecular techniques for identifying iDNA, including PCR‐only (Lee et al., 2015), qPCR (Kampmann et al., 2017), Sanger sequencing (Schnell et al., 2012) or shot‐gun sequencing of individuals (Gómez & Kolokotronis, 2016) and high‐throughput sequencing (HTS) of amplicon pools (Calvignac‐Spencer, Leendertz, et al., 2013). Here, we independently confirm the observation by Schnell et al. (2018) that sample throughput can be successfully maximized by pooling individual DNA extracts before screening for iDNA, a technique that has allowed us to conduct such a comprehensive investigation of the area. By only sequencing leech pools that contained successfully amplified DNA, we were able to maximize cost‐effectiveness in our study. At the same time, however, pooling represents a trade‐off; by not determining the feeding behaviour of individual leeches within the pool, we may underestimate the importance of some mammalian prey. Discerning the consequences of pooling for iDNA detection in leeches is an important consideration when applying these technologies in conservation monitoring programmes.

### 4.4 Leeches in human‐modified forests

We sampled leeches in a degraded human‐modified landscape, which is becoming a typical ecosystem in South‐East Asia and has been associated with a different mammalian composition compared to primary rainforest (Wearn et al., 2017). Using iDNA, we found a 30%–40% overlap in genera detected compared to two camera trapping studies which were conducted at the same field site (Deere et al., 2017; Wearn et al., 2017). With a much shorter field sampling campaign compared to the comprehensive camera trapping of these two studies, this highlights the potential of the iDNA method as a rapid and complementary sampling tool. Detecting diversity in leech diets is affected by many factors. One such factor being the restriction of terrestrial leeches to areas with high humidity as a consequence of their evolutionary history (Borda & Siddall, 2004). As we found in this study, that some heavily degraded and open forest plots yielded no leeches on multiple visits. While we do not know how leech populations will be affected with increasing land‐use change, temperature increases and humidity decreases as forests are fragmented (Hardwick et al., 2015) and logging has already been shown to affect a wide range of invertebrates (Ewers et al., 2015). As such, it is likely that land‐use change will have a detrimental effect on terrestrial leech populations. It might be beneficial therefore to test alternative invertebrate samplers, such as blowflies, which are found in a greater variety of habitats (Calvignac‐Spencer, Merkel, et al., 2013).

One observable consequence of sampling leeches in logged forests was the high proportion of human DNA detected in our samples (45% of samples with >10% of total copy number). Human activity is high in degraded forests (also see Weiskopf et al., 2017) especially around the SAFE project field site, where there are semipermanent forestry and oil‐palm settlements scattered throughout the landscape. Thus, we would assume human blood meals are sustaining leech populations in degraded landscapes.

## 5 CONCLUSIONS

It is important to understand how invertebrate behaviours will introduce biases and affect our biodiversity estimates from iDNA monitoring. By exploring the diets of two congeneric leech species, we found that they are not equal in their ability to detect mammals. We would recommend that in the forests in Borneo, where the focal leeches are co‐occurring, *H. picta* is the more effective iDNA sampler for both molecular and behavioural reasons. However, in habitats where only *H. sumatrana* is found, iDNA recovered from this species should be sufficient to detect common mammals. The lack of detection of small mammal families from the diets of both species in Borneo shows how little we know about terrestrial leech behaviours. With this study, we emphasize the need to understand as much of the ecology as possible for the iDNA invertebrate sampler of choice and how the species interacts with the environment. We would recommend more studies, such as this one, especially if the ultimate goal is for conservation monitoring. But for these congeneric leeches, this study adds to our understanding of their feeding ecology for which previously there was little known and puts us a step closer to utilizing iDNA in future monitoring programmes. Finally, few iDNA studies have considered the potential impacts of over‐harvesting invertebrate sampler species, and the conservation implications of this are not known (Schnell, Sollmann, et al., 2015). In general, the role of leeches in the wider ecosystem is poorly understood; thus, we advocate to reduce the numbers extracted, in areas of Borneo where *H. picta* and *H. sumatrana* co‐occur, only *H. picta* is needed to sample the community of mammals.

## CONFLICT OF INTERESTS

The authors have no conflict of interests.

## AUTHOR CONTRIBUTIONS

S.J.R. and R.D. designed the study. R.D. collected and analysed the data. M.T.P.G., I.B.S., K.B. provided laboratory, facilities, protocols and bioinformatics pipelines. G.V. generated new sequences and H.B. facilitated fieldwork. E.C. contributed to laboratory procedures and data processing. R.D. and S.J.R. wrote the study with critical input from all authors.

## Data Availability

All 16S sequences generated for this study are available on GenBank, Accession nos: MG996889–MG996900. All data are available on the NERC Environmental Information Data Centre (EIDC), https://doi.org/10.5285/3affed0d-fe6f-4916-89e3-e672639191e5.
