# Supplementary material for: Using metabarcoding to compare the suitability of two blood‐feeding leech species for sampling mammalian diversity in North Borneo
Source: Mol Ecol Resour. 2018 Oct 16;19(1):105–17. doi: 10.1111/1755-0998.12943 (PMC7379310; doi:10.1111/1755-0998.12943)
Supplement: Supplementary file 1 [file MEN-19-105-s001.pdf]

MOLECULAR ECOLOGY RESOURCES

Supplemental Information for:

Using metabarcoding to compare the suitability of two blood-feeding leech species for sampling mammalian diversity in North Borneo

Rosie Drinkwater, Ida Bærholm Schnell, Kristine Bohmann, Henry Bernard, Géraldine Veron, Elizabeth Clare, M. Thomas P. Gilbert, Stephen J. Rossiter

Table S1. Description of the novel sequences generated for this study with the tissue origin and accession number from NCBI GenBank. MNHM = Muséum national d'Histoire naturelle, Paris, FR; USNM = Smithsonian National Museum of Natural History, Washington, USA; BZM = Museum fur Naturkunde, Berlin, GER; ROM = Royal Ontario Museum, Toronto, CA; NHM = Natural History Museum, London, UK; FMHN = Field Museum, Chicago, USA. The Chinese ferret badger sequence was used as an alternative closely related sister species to the Bornean ferret badger for which there was no sequence available.

**Common name**	**Family**	**Genus**	**Species**	**Origin**	**Institution**	**Accession number**
Clouded leopard	Felidae	*Neofelis*	*nebulosa*	Ménagerie, MNHN	MNHN	MG996889
Short tailed mongoose	Herpestidae	*Urva*	*brachyura*	Malaysia, Borneo, Sarawak	USNM	MG996890
Collared mongoose	Herpestidae	*Urva*	*semitorquata*	Borneo	BZM	MG996891
Chinese ferret badger	Mustelidae	*Melogale*	*moschata*	Vietnam	ROM	MG996892
Malay weasel	Mustelidae	*Mustela*	*nudipes*	Malaysia	MNHN	MG996893
Banded linsang	Prionodontidae	*Prionodon*	*linsang*	Cincinnati Zoo	MNHN	MG996894
Small-toothed palm civet	Viverridae	*Arctogalidia*	*trivirgata*	Ménagerie, MNHN	MNHN	MG996895
Hose's civet	Viverridae	*Diplogale*	*hosei*	Borneo	NHM	MG996896
Banded civet	Viverridae	*Hemigalus*	*derbyanus*	Indonesia, Borneo	MNHN	MG996897
Common palm civet	Viverridae	*Paradoxurus*	*hermaphroditus*	Indonesia, Borneo	ROM	MG996898
Malay civet	Viverridae	*Viverra*	*tangalunga*	Philippines	FMNH	MG996899
Common treeshrew	Tupaiidae	*Tupaia*	*glis*	Thailand	MNHN	MG996900

MOLECULAR ECOLOGY RESOURCES

Table S2. List of species included in the database which were additional to the Bornean mammals.

Common name	Species name	Reason for inclusion
African civet	*Civettictis civetta*	Only one record for Malay civet, sister taxa
Indian crested porcupine	*Hystrix indica*	Only one record for Malay porcupine, no records for thick-spined porcupine, sister taxa
Giraffe	*Giraffa camelopardalis*	Used as positive control, divergent to Bornean species
Domestic cattle	*Bos taurus*	Potentially present in the area, human associated
Malaysian field rat	*Rattus tiomanicus*	Potentially present in the area, human associated
Brown rat	*Rattus norvegicus*	High probability species is present, human associated
House rat	*Rattus rattus*	High probability species is present, human associated
House mouse	*Mus musculus*	High probability species is present, human associated
Domestic dog	*Canis lupis familiaris*	Presence confirmed at the field site
Domestic cat	*Felis sylvestris*	Presence confirmed at the field site
Bent toe gecko	*Gehyra mutilata*	Representative common gecko sequence; potential blood meal
Asian house gecko	*Hemidactylus frenatus*	Representative common gecko sequence; potential blood meal
Chicken	*Gallus gallus*	Representative common fish sequence; presence confirmed at the field site; potential blood meal
Monitor lizard	*Varaus salavator*	Presence confirmed at the field site; potential blood meal
Freshwater fish	*Leprobarbus hovenii*	Representative common fish sequence

MOLECULAR ECOLOGY RESOURCES

Table S3. Model summaries for the candidate models testing variation in overall count of detections.

Model structure	AIC	DF	ΔAIC	Residual deviance	Adj - R^2
Habitat + Leech + Site + Habitat:Leech + Site:Leech	307.25	11	12.8	37.5	0.545
Habitat + Leech + Site + Site:Leech	307.25	11	12.8	37.5	0.546
Habitat + Leech + Site + Habitat:Leech	302.60	8	8.2	38.8	0.5094
Habitat + Leech + Site	301.25	7	6.8	39.5	0.4983
Leech + Habitat	295.65	3	0	42.6	0.3086
Leech + Site	301.25	7	6.8	39.5	0.4983
Leech	294.41	2	1.2	41.9	0.2473

MOLECULAR ECOLOGY
RESOURCES

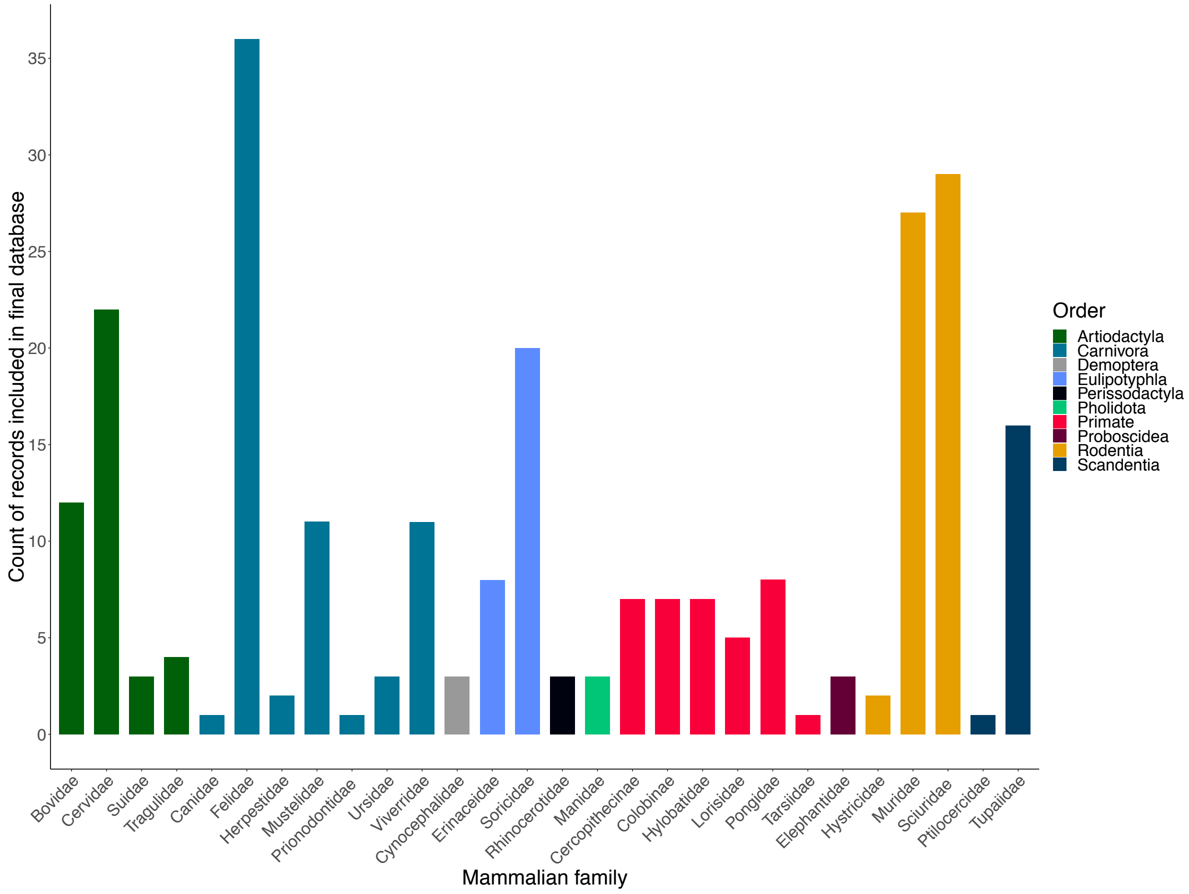

Figure S1. Summary of number of records included in the 16S rRNA reference database. Grouped into mammalian family and coloured by mammalian order.

MOLECULAR ECOLOGY RESOURCES

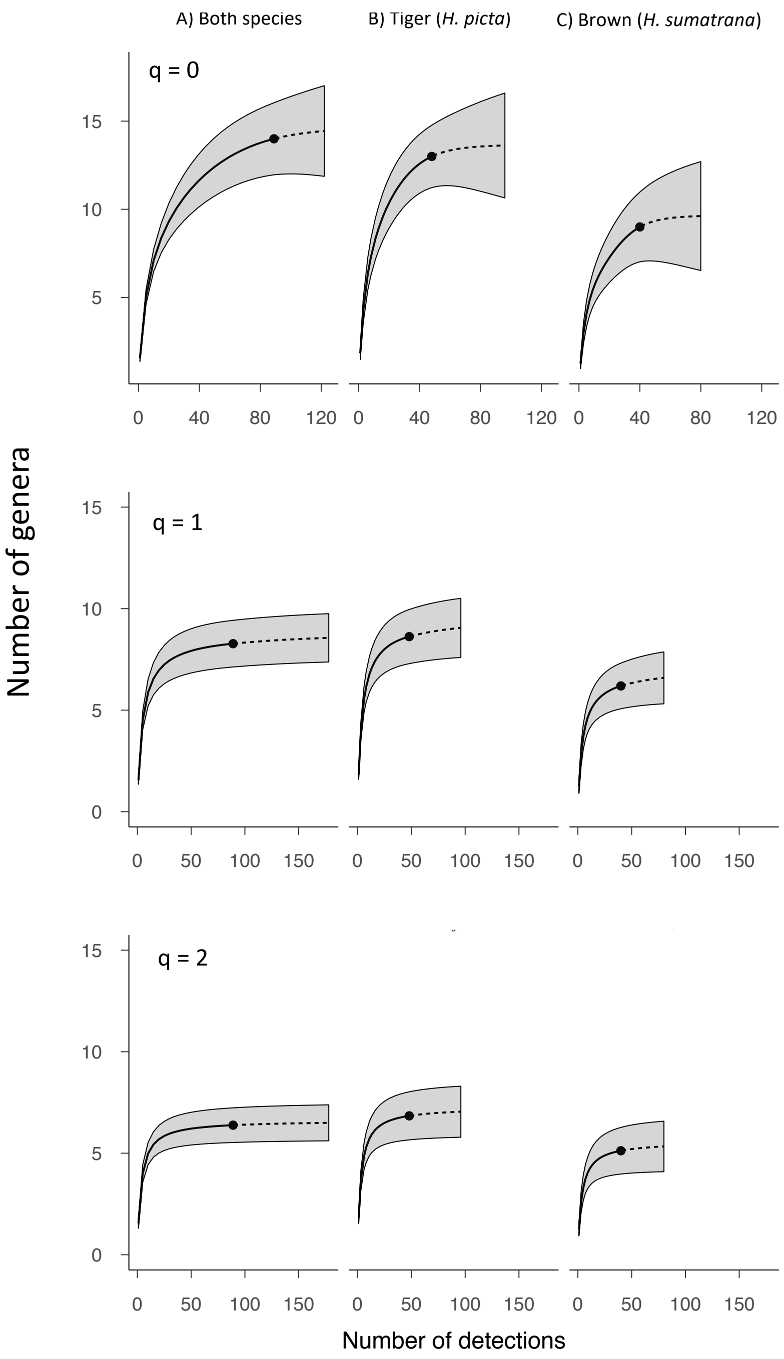

Figure S2. Diversity accumulation curves with 95% confidence intervals. For all samples (A), *H. picta* only (B) and *H. sumatrana* (C) – at three orders of q.
